# Abnormal neural activities of directional brain networks in patients with long-term bilateral hearing loss

**DOI:** 10.18632/oncotarget.20361

**Published:** 2017-08-19

**Authors:** Long-Chun Xu, Gang Zhang, Yue Zou, Min-Feng Zhang, Dong-Sheng Zhang, Hua Ma, Wen-Bo Zhao, Guang-Yu Zhang

**Affiliations:** ^1^ Department of Radiology, Taishan Medical University, Taian 271016, Shandong Province, China; ^2^ Department of Radiology, Affiliated Hospital of Taishan Medical University, Taian 271000, Shandong Province, China; ^3^ Department of Otorhinolaryngology and Head-Neck Surgery, Affiliated Hospital of Taishan Medical University, Taian 271000, Shandong Province, China; ^4^ Department of Medical Information Engineering, Taishan Medical University, Taian 271016, Shandong Province, China

**Keywords:** multivariate regression model, causal connectivity, the virtual brain, bilateral hearing loss, cognitive decline

## Abstract

The objective of the study is to provide some implications for rehabilitation of hearing impairment by investigating changes of neural activities of directional brain networks in patients with long-term bilateral hearing loss. Firstly, we implemented neuropsychological tests of 21 subjects (11 patients with long-term bilateral hearing loss, and 10 subjects with normal hearing), and these tests revealed significant differences between the deaf group and the controls. Then we constructed the individual specific virtual brain based on functional magnetic resonance data of participants by utilizing effective connectivity and multivariate regression methods. We exerted the stimulating signal to the primary auditory cortices of the virtual brain and observed the brain region activations. We found that patients with long-term bilateral hearing loss presented weaker brain region activations in the auditory and language networks, but enhanced neural activities in the default mode network as compared with normally hearing subjects. Especially, the right cerebral hemisphere presented more changes than the left. Additionally, weaker neural activities in the primary auditor cortices were also strongly associated with poorer cognitive performance. Finally, causal analysis revealed several interactional circuits among activated brain regions, and these interregional causal interactions implied that abnormal neural activities of the directional brain networks in the deaf patients impacted cognitive function.

## INTRODUCTION

A meta-analysis in adults [1] proposed a relationship between the hearing impairment and cognitive changes. Accumulating evidences have also pointed to a link between hearing impairment and cognitive decline [2–8]. These evidences mainly resulted from the clinical investigations. In those studies, neuropsychological testing and pure tone audiometry revealed a link between hearing loss and poorer cognitive performance [2–6]. However, causal mechanisms of the link between hearing loss and cognitive decline are not entirely clear. Investigations of the causal mechanisms can provide some implications for prevention, rehabilitation of hearing impairment and healthy policy [8]. Four hypotheses have been proposed to explain the causal mechanisms of this link. Cognitive load on perception hypothesis, according to this view, cognitive decline increases the load on perception of sound and weakens hearing ability, there is little evidence supporting this hypothesis [8, 9]; Information degradation hypothesis [10], which assumes that impaired auditory input contributes to more extensive use of cognitive resources to compensate for the loss of speech information, thereby reducing the cognitive resources that are available for other cognitive tasks, resulting in an impairment in cognitive performance [8, 11]; sensory deprivation hypothesis [6, 8–10, 12] proposes that long-term auditory deprivation leads to functional reorganization of cerebral cortex and reallocation of resources. As a result, these changes cause the impairment of cognitive abilities; common cause hypothesis posits that an age-related neural degeneration contributes to the declines in hearing and cognitive functions [9, 13]. In this study, we investigated changes of neural activities of brain regions in patients with long-term bilateral hearing loss by utilizing the virtual brain. Our objective was to provide some insights into causal mechanisms of the link between cognitive decline and hearing loss. The virtual brain is a model of directional brain network, every node of the network denotes a Brodmann area (BA). Neural activities of each brain region in the virtual brain can be predicted by using a multivariate regression method. The directional brain network is constructed by utilizing an effective connectivity method [14] based on resting-state functional magnetic resonance (fMRI) data of participants.

## RESULTS

### Changes of brain region activations in patients with bilateral hearing loss

**Figure 1:**
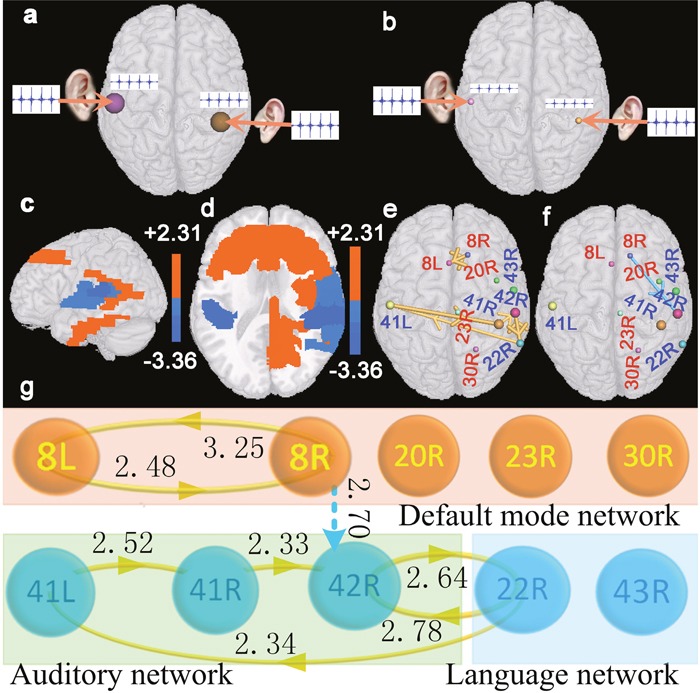
Results of brain region activations (two-sample t-test, p<0.05, FDR corrected) **(a)** denotes a diagram of the auditory stimulating signal exerted to bilateral ears of the virtual digital brain constructed based on resting-state fMRI data of the normal hearing subjects. **(b)** denotes a diagram of the auditory stimulating signal exerted to bilateral ears of the virtual digital brain constructed based on resting-state fMRI data of patients with hearing loss. **(c** and **d)** denote changes of brain region activations in patients with hearing loss as compared with normal hearing subjects. Red brain regions indicate that patients present enhanced activations in these areas, and blue regions indicate that patients present weakened activations in those areas. **(e** and **f)** denote interregional causality connections. The size of the sphere indicates the difference of the brain region activation between patients and normal hearing subjects, and the diameter of the bar denotes the strength of the interregional causality connectivity. The gold bar denotes the synchronous causality connectivity, and the light blue bar denotes the asynchronous causality connectivity. The direction of the arrow denotes the direction of causality connectivity. **(g)** is a graph corresponding to (e and f). The light blue dot line corresponds to the light blue bar. The number beside each line or bar indicates the strength of interregional causality connectivity. Abbreviations see Table 1 for details.

Firstly, we constructed the individual specific virtual brain using resting-state fMRI data of each participant. Then we generated auditory stimulating signals, which were composed of the sum of sines and cosines of various frequencies with range from 500Hz to 2000Hz. To simulating a real environment, we presumed that the auditory signals were decayed in the transmission process from the cochlea to the primary auditory cortex (BA 41), and the weakened amplitudes were different according to the normal hearing subjects and the deaf patients respectively (Figure 1a and 1b). The generated auditory stimulating signals were exerted to the virtual brains through bilateral ears, and brain regions were activated by the stimulating signals. The statistical method was used to analyze brain region activations. The false discovery rate (FDR) was utilized to correct the statistical analysis results.

**Table 1:** Indexes of BAs defined in the study

Indexes	Brain regions	Indexes	Brain regions
1R, 3R	Right primary somatosensory cortex	2L, 3L	Left primary somatosensory cortex
5L, 7L	Left somatosensory association cortex	10L	Left anterior prefrontal cortex
8L	Left dorsal frontal cortex	8R	Right dorsal frontal cortex
9L	Left dorsolateral prefrontal cortex	9R	Right dorsolateral prefrontal cortex
13L	Left insular cortex	13R	Right insular cortex
20L	Left inferior temporal gyrus	20R	Right inferior temporal gyrus
21L	Left middle temporal gyrus	21R	Right middle temporal gyrus
22L	Left superior temporal gyrus	22R	Right superior temporal gyrus
30R	Right cingulate cortex	23R	Right ventral posterior cingulate cortex
31L	Left dorsal posterior cingulate cortex	31R	Right dorsal posterior cingulate cortex
32L	Left dorsal anterior cingulate cortex	32R	Right dorsal anterior cingulate cortex
36L	Left parahippocampal cortex	36R	Right parahippocampal cortex
37L	Left fusiform gyrus	37R	Right fusiform gyrus
38L	Left temporopolar area	38R	Right temporopolar area
39L	Left angular gyrus	39R	Right angular gyrus
40L	Left supramarginal gyrus	40R	Right supramarginal gyrus
41L	Left primary auditory cortex	41R	Right primary auditory cortex
42L	Left primary auditory cortex	42R	Right primary auditory cortex
43L	Left subcentral area	43R	Right p subcentral area
44L	Left inferior frontal cortex	44R	Right inferior frontal cortex
45L	Left inferior frontal cortex	45R	Right inferior frontal cortex
46L	Left dorsolateral prefrontal cortex	46R	Right dorsolateral prefrontal cortex
47L	Left inferior prefrontal gyrus	47R	Right inferior prefrontal gyrus

The results are shown in Figure 1. Compared with normal hearing subjects, two-sample t-test revealed that the patients with bilateral hearing loss presented significantly increased neural activities (two-sided, p<0.05, FDR corrected) in the superior medial frontal cortex (BAs 8L and 8R), the right cingulate cortex (BA 30R), the right posterior cingulate cortex (BA 23R), and the right inferior temporal gyrus (BA 20R) in the default mode network (DMN); decreased neural activities in the primary auditory cortex (BAs 41L, 41R, and 42R) of the auditory network, the right superior temporal gyrus (BA 22R) and subcentral area (BA 43R) of the language network.

**Figure 2:**
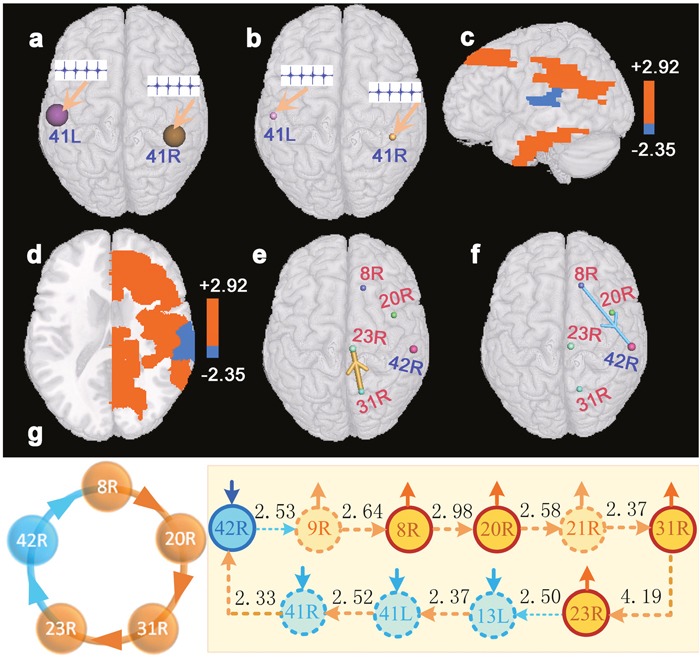
Results of brain region activations evoked by the stimulating signals exerted to BAs 41L and 41R (two-sample t-test, p<0.05, FDR corrected) **(a)** denotes a diagram of the auditory stimulating signals exerted to BAs 41L and 41R of the virtual digital brain constructed based on resting-state fMRI data of normal hearing subjects. **(b)** denotes a diagram of the same auditory stimulating signals exerted to BAs 41L and 41R of the virtual digital brain constructed based on resting-state fMRI data of patients with hearing loss. **(c** and **d)** denote changes of brain region activations in patients with hearing loss as compared with normal hearing subjects. Red brain regions denote that patients present enhanced activations in these areas, and blue regions indicate that patients present weakened activations in those areas. **(e** and **f)** denote interregional causality connections. The size of the sphere indicates the difference of the brain region activation between patients and normal hearing subjects, and the diameter of the bar denotes the strength of the interregional causality connectivity. The gold bar denotes the synchronous causality connectivity, and the light blue bar denotes the asynchronous causality connectivity. The direction of the arrow denotes the direction of causality connectivity. **(g)** is a graph of causal connectivity among BAs 42R, 8R, 20R, 31R, and 23R. The light blue dot line or solid arc line denote asynchronous causal connectivity, and the orange dot line or solid arc line denote synchronous causal connectivity. The direction of the arrow denotes the direction of causality connectivity, and the number beside each line indicates the strength of interregional causality connectivity. The solid short arrows denote changes of the strengths of activations. Abbreviations see Table 1 for details.

To further verify the influences of long-term bilateral hearing loss on cerebral cortices, we exerted directly the auditory stimulating signals to BAs 41L and 41R (Figure 2a and 2b), and investigated changes of brain region activations in patients with hearing loss. Two-sample t-test revealed that patients with bilateral hearing loss presented significantly reduced brain region activation (two-sided, p<0.05, FDR corrected) in BA 42R but enhanced activations in BAs 8R, 20R, 23R, and 31R as compared with normal hearing subjects (Figure 2c and 2d).

### Causal connections among brain regions

**Figure 3:**
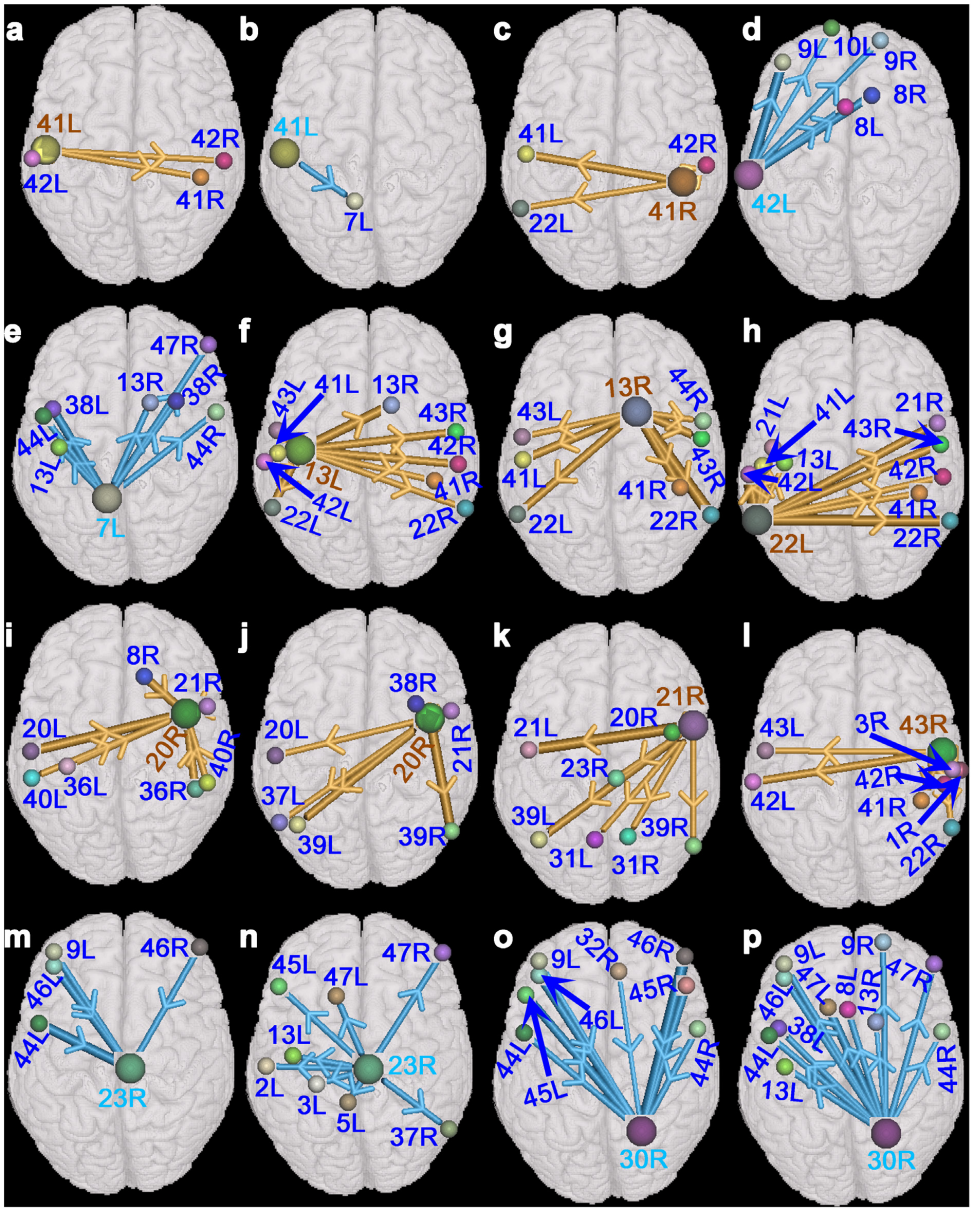
A diagram of input and output causal connections (two-sided, p<0.05, FDR corrected) **(a, c, f, g, h, j, k, and l)** denote the graphs of synchronous output causal connectivity of BAs 41L, 41R, 13L, 13R, 22L, 20R, 21R, and 43R, respectively. **(b, d, e, n, and p)** denote the graphs of asynchronous output causal connectivity of BAs 41L, 42L, 7L, 23R, and 30R, respectively. **(i)** denotes a graph of synchronous input causal connectivity of BA 20R. **(m** and **o)** denote the graphs of asynchronous input causal connectivity of BAs 23R and 30R, respectively. The gold bar denotes the synchronous causality connectivity, and the light blue bar denotes the asynchronous causality connectivity. The direction of the arrow denotes the direction of causality connectivity, and the diameter of the bar denotes the strength of the interregional causality connectivity. Abbreviations see Table 1 for details.

We studied the interregional synchronous or asynchronous entropy connections (causality connections) among those brain regions that presented significant changes (two-sided, p<0.0005, FDR corrected) of activated strengths in the deaf patients utilizing causal analysis method [14]. Results indicated that BA 8R presented asynchronous causality connections with BA 42R. Synchronous causality connections were found between BAs 8R and 8L, together with between BAs 42R and 22R. In addition, synchronous causality connections were also found among other brain regions (Figure 1e-1g). We also noted that Figure 2e-2g presented a synchronous causal connectivity between BAs 31R and 23R but an asynchronous causal connectivity between BAs 8R and 42R. Figures 2 and 3 showed only parts of interregional causal connections. To analyze causal mechanism of cognitive decline, we further studied input and output causal connections of some brain regions (two-sided, p<0.05, FDR corrected), including BAs 41L, 41R, 42L, 7L, 13L, 13R, 22L, 20R, 21R, 43R, 23R, and 43R (Figure 3). BA 41L presented synchronous output causal connections with BAs 41R, 42L, and 42R (Figure 3a) but asynchronous output causal connectivity with BA 7L (Figure 3b); BA 41R presented synchronous output causal connections with BAs 22L, 41L, and 42R (Figure 3c); BA 42L presented asynchronous output causal connections with BAs 8L, 8R, 9L, 9R, and 10L (Figure 3d); BA 7L presented asynchronous output causal connections with BAs 13L, 13R, 38L, 38R, 44L, 44R, and 47R (Figure 3e); BA 13L presented synchronous output causal connections with BAs 13R, 22L, 22R, 41L, 41R, 42L, 42R, 43L, and 43R (Figure 3f); BA 13R presented synchronous output causal connections with BAs 22L, 22R, 41L, 41R, 43L, 43R, and 44R (Figure 3g); BA 22L presented synchronous output causal connections with BAs 13L, 21L, 21R, 22R, 41L, 41R, 42L, 42R, and 43R (Figure 3h); BA 20R presented synchronous input causal connections with BAs 8R, 20L, 21R, 36L, 36R, 40L, and 40R (Figure 3i) but synchronous output causal connections with BAs 20L, 21R, 37L, 38R, 39L, and 39R (Figure 3j); BA 21R presented synchronous output causal connections with BAs 20R, 21L, 23R, 31L, 31R, 39L, and 39R (Figure 3k); BA 43R presented synchronous output causal connections with BAs 1R, 3R, 22R, 41R, 42L, 42R, and 43L (Figure 3l); BA 23R presented asynchronous input causal connections with BAs 9L, 44L, 46L, and 46R (Figure 3m) but asynchronous output causal connections with BAs 2L, 3L, 5L, 13L, 37R, 45L, 47L, and 47R (Figure 3n); BA 30R presented asynchronous input causal connections with BAs 9L, 32R, 44L, 44R, 45L, 45R, 46L, and 46R (Figure 3o) but asynchronous output causal connections with BAs 8L, 9L, 9R, 13L, 13R, 38L, 44L, 44R, 46L, 47L, and 47R (Figure 3p).

### Causal circuits among brain regions

**Figure 4:**
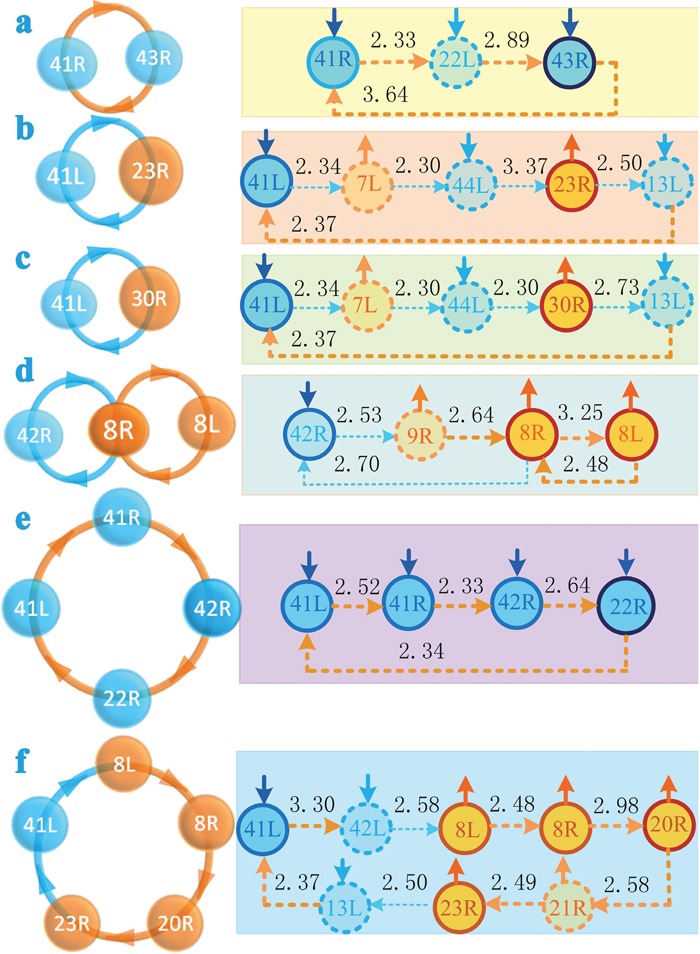
A diagram of causal circuits among brain regions (two-sided, p<0.05, FDR corrected) **(a)** denotes a causal circuit between BAs 41R and 43R. **(b)** denotes a causal circuit between BAs 41L and 23R. **(c)** denotes a causal circuit between BAs 41L and 30R. **(d)** denotes the causal circuits among BAs 42R, 8R, and 8L. **(e)** denotes a causal circuit among BAs 41L, 41R, 42R, and 22R. **(f)** denotes a causal circuit among BAs 41L, 8L, 8R, 20R, and 23R. The light blue dot line or solid arc line denote asynchronous causal connectivity, and the orange dot line or solid arc line denote synchronous causal connectivity. The direction of the arrow denotes the direction of causality connectivity, and the number beside each line indicates the strength of interregional causality connectivity. The solid short arrows denote changes of the strengths of activations. Abbreviations see Table 1 for details.

We found several causal circuits among those brain regions whose activity strengths presented significant changes through analyzing Figures 1, 2, and 3. As shown in Figure 4a, there existed a causal circuit between the right primary auditory cortex (BA 41R) and the right subcentral area (BA 43R). This causal circuit indicated that reduced auditory response induced by hearing loss in BA 41R led to decreased activation in BA 22L, which caused significantly decreased activation in BA 43R, and the change of activated strength in BA 43R further weakened auditory response of BA 41R through a synchronous causal connectivity. There existed also a causal circuit between the left primary auditory cortex (BA 41L) and the right ventral posterior cingulated cortex (BA 23R) that is widely known as a central node in the DMN. This circuit implied that decreased activation of BA 41L contributed to increased activity in BA 7L but decreased activation in BA 44L, and finally resulted in enhanced activation in BA 23R. On the other hand, increased neural activity in BA 23R weakened also auditory response of BA 41L through the left insular cortex (BA 13L) (Figure 4b). In addition, we found also a similar causal interactive circuit between BAs 41L and 30R (Figure 4c). The right primary auditory cortex (BA 42R) presented also a causal circuit with the right dorsal frontal cortex (BA 8R), and this circuit revealed a causal interaction that decreased activity strength in BA 42R contributed to enhanced neural activity in BA 8R through the right dorsolateral prefrontal cortex (BA 9R), and this change of BA 8R also suppressed neural activity of BA 42R. A causal interaction was also observed between BAs 8R and 8L (Figure 4d). The causal circuit among BAs 41L, 41R, 42R, and 22R (Figure 4e) indicated that reduced responses in auditory cortices caused the decline of activated strength in BA 22R, which further weakened auditory response of BA 41L. The causal circuits among BAs 41L, 8L, 8R, 20R, and 23R (Figure 4f), as well as among BAs 42R, 8R, 20R, 23R, and 31R (Figure 2g) indicated that reduced responses in auditory cortices contributed to enhanced brain region activations in the DMN, and these activations also depressed response of auditory cortices.

### Neuropsychological tests

Mini-mental state examination (MMSE) and Wechsler adult intelligence test [15] were used to assess the cognitive abilities of the participants. The results of tests are shown in Table 2. The patients with bilateral hearing loss presented poorer performance scores in all neuropsychological tests, especially, significant differences were observed in MMSE and stroop color-word test A. In addition, recall and language processing scores also displayed significant differences.

**Table 2:** Summary of behavior tests

Tests		Hearing loss (11)	Normal (10)	Statistic(df)	P
Mini-mental state examination(MMSE)		26.73±1.6	28.9±1.1	T=-3.56(19)	0.002^*^
	I Orientation	9.73±0.65	10±0.0	T=-1.40(10)^**^	0.19
	II Registration	2.91±0.30	3.0±0.0	T=-1.0(10)^**^	0.34
MMSE	III Attention and calculation	4.82±0.40	5.0±0.0	T=-1.49(10)^**^	0.17
	IV Recall	2.0±1.0	2.80±0.42	T=-2.34(19)	0.03^*^
	V Language processing	6.63±0.81	7.4±0.84	T=-2.12(19)	0.048^*^
	VI Complex commands	0.64±0.50	0.80±0.42	T=-0.80(19)	0.43
Digit symbol substitution test		38.5±14.0	44.0±13.7	T=-0.92(19)	0.370
Verbal fluency		27.3±5.7	32.8±6.4	T=-2.08(19)	0.051
Forward digit spans		7.1±0.83	7.6±0.97	T=-1.30(19)	0.210
Backward digit spans		4.4±0.50	5.1±1.3	T=-1.76(19)	0.095
Trail making test part A		52.5±16.0	43.3±9.3	T=1.64(16.3)^**^	0.121
Trail making test part B		151.0±44.3	123.4±43.3	T=1.44(19)	0.166
Stroop color–word test A		29.5±7.0	23.5±3.2	T=2.54(14.4)^**^	0.023^*^
Stroop color–word test B		46.2±13.7	37.5±6.3	T=1.84(19)	0.082
Stroop color–word test C		85.6±22.9	68.6±16.1	T=1.95(19)	0.066

Note.—Data are mean ± standard deviation.^*^: p<0.05 is considered as a significant difference. ^**^: homogeneity test (Levene's test), p<0.05, revised t-test.

### Correlation analysis

We investigated the relationships among the hearing levels, neuropsychological testing scores, and activity strengths of significantly activated brain regions by using Pearson's correlation analysis. Cognitive performance scores in MMSE and recall presented strongly negative correlation with the left hearing levels of participants (r=-0.553, p=0.009; r=-0.528, p=0.014); on the contrast, testing scores in stroop color-word test A (SCWTA) presented positive correlation with the left hearing levels (r=0.549, p=0.01). Both activated strengths in BAs 41L and 41R displayed significant negative correlations with SCWTA testing scores (r=-0.508, p=0.019; r=-0.524, p=0.015). Lower SCWTA performance scores and higher MMSE testing scores indicate better cognitive abilities. Activated strengths in BA 41L was positively related with language processing scores (r=0.436, p=0.048), similarly, Activated strengths in BA 41R was positively related with MMSE testing scores (r=0.490, p=0.024). Activated strengths in BAs 41L, 41R, and 42R presented strongly positive correlations with the left hearing levels (r=-0.662, p=0.001; r=-0.778, p<0.0001; r=-0.711, p<0.001) and the right hearing levels of participants (r=-0.636, p=0.002; r=-0.792, p<0.0001; r=-0.663, p=0.001).

## DISCUSSION

In this study, we investigated changes of neural activities of directional brain networks in patients with long-term bilateral hearing loss. Our main findings are described as follows. Compared with normal hearing subjects, patients with long-term bilateral hearing loss presented weaker brain region activations in the primary auditory cortex (including BAs 41L, 41R, and 42R) and the language network; enhanced neural activities in the DMN. In addition, we also found some significant correlations among the hearing levels, neuropsychological testing scores, and activated strengths in the primary auditory cortex. Poorer cognitive performance scores were always associated with higher hearing levels and weaker activated strengths in BAs 41L and 41R. Previous study also demonstrated that hearing loss was independently associated with poor cognitive performance scores [2, 3, 16].

### Causal interaction between the auditory and language networks

We also found reduced neural activities in BAs 22R and 43R of the language network. Moreover, neuropsychological tests revealed a significant difference in language processing scores between the deaf patients and the controls. Previous studies [17, 18] indicated that the right superior temporal gyrus (BA 22R) and the right subcentral area (BA 43R) were associated with language processes and social cognition. However, Pearson's correlation analysis failed to find a significant relationship between language processing scores and activated strengths in the language network. One explanation is that these two areas in the language network affect the deaf patients’ speech processing abilities through an indirect manner. As shown in this study, there existed several causal circuits among BAs 41L, 22R, 41R, 43R, and these causal interactions indicated that reduced activity strengths in the language network resulted in decreased auditory cortical responses through a causal feedback mechanism. Weakened auditory responses caused loss of speech information and resulted in the decline of language processing abilities of the deaf patients. This explanation is consistent with our finding that language testing scores presented a significant correlation with activated strengths in BA 41L.

### Causal interaction between the auditory and default mode networks

Several brain regions associated with cognitive processes in the DMN displayed enhanced activations. These areas included the dorsal frontal cortex (BA 8) involving the management of uncertainty [19]; the right inferior temporal gyrus (BA 20R) that is responsible for recognizing patterns, faces, and objects [20]; the right cingulate cortex (BA 30R) that is involved with processing emotion [21]; the right posterior cingulate cortex (BAs 23R and 31R) that has also been firmly linked to emotional salience and autobiographical memory retrieval [22, 23]. Our finding is consistent with previous study results [24, 25] that the deaf patients presented enhanced functional connectivity in the DMN. Commonly, enhanced neural activities in the DMN are thought to be an improvement of cognitive processing function. However, we failed to find a significant relationship between activated strengths of these brain regions and neuropsychological testing scores based on Pearson's correlation analysis. Therefore, these abnormal changes in the DMN did not directly affect the cognitive abilities of patients with bilateral hearing loss. Whereas the neuropsychological tests indicated that the deaf patients presented a significant decline of cognitive abilities. What are the causal mechanisms between changes of the DMN and the cognitive decline? To address this issue, we investigated the causal interaction between the auditory and default mode networks. We found that there existed some causal interacting circuits among brain regions in the auditory and default mode networks. Hearing loss contributed to reduced auditory responses in the primary auditory cortex, and these decreased responses were further diminished through synchronous causal interactions among brain regions within the auditory network. On the other hand, reduction of auditory cortical responses also led to the decline of inhibitive ability of the auditory network for the DMN through asynchronous interregional interactions. As a result, these changes contributed to enhanced brain region activations in the DMN, which suppressed auditory cortical perception and processing abilities through an asynchronous causal feedback mechanism. These interactions between the auditory network and the DMN ultimately resulted in cognitive decline of patients with bilateral hearing loss. In a word, Long-term hearing loss not only affects the perception of patients for sound but also contributes to changes of the brain functional networks. These changes result in the decline of cognitive abilities (including language processing) through an indirect causal interacting mechanism. Therefore, our results indicated that long-term hearing loss contributed to increased neural activities in the DMN, and these abnormal changes resulted in the decline of the deaf patients’ cognitive abilities through inhibiting the response of auditory cortices.

It is worth noting that patients with bilateral hearing loss presented more changes in the right cerebral hemisphere than those in the left. Additionally, the left hearing levels presented strongly correlation with MMSE and SWCA testing scores. One possible reason is that long-term hearing loss contributes to complex causal interactions among brain regions, and these causal circuits lead to more changes in the right cerebral hemisphere than in the left. Another possible reason is that the right cerebral hemisphere is more sensitive to hearing loss than the left. Transmission of auditory signals is lateralized to the contralateral hemisphere. Therefore, this finding suggests that left hearing loss may affect the cerebral cortex more than the right hearing loss does, as shown in previous study [25].

In a word, long-term causal interactions were main reasons that contributed to the decline of the deaf patients’ cognitive abilities. Our findings imply that early clinical interference is important for the deaf patients, especially, left cochlear implant might have more advantages than the right for patients that had bilateral post-lingual deafness.

## MATERIALS AND METHODS

### Subjects

The protocol of this prospective study was approved by the institutional Ethics Committee of Taishan Medical University, and all participants signed informed consent forms prior to the experiment. A total of 21 subjects (11 patients with long-term bilateral hearing loss and 10 subjects with normal hearing; the duration of hearing loss: 3-55 years, average=20.3±15.7) were recruited from Taian. Specially, 10 patients had long-term bilateral hearing loss with tinnitus. Hearing loss was acquired due to traumatism in one patient, deafness was caused by infection in two patients, and hearing loss in others was unknown cause. All were right-handed and none of them had a history of medical, strokes/cerebrovascular ischemia. Demographic characteristics of participants are shown in Table 3. Hearing level (HL) was defined as a speech-frequency pure tone average of thresholds at 0.25, 0.5, 1.0 and 2 kHz. There was no significant difference between the left and right sided ears (p>0.05). There was significant difference of hearing levels between the deaf group and the controls (p<0.0001). All deaf subjects had post-lingual deafness. There were no significant differences for age, education and sex between the hearing loss group and the controls (p>0.05). Cognitive abilities of participants were measured by using the neuropsychological tests [25].

**Table 3:** Demographic characteristics of participants

Group (n)	Hearing loss (11)	Normal (10)	Statistic(df)	P
Age (years)	52.8±8.5	52.5±7.0	T=0.119(19)	0.906
Education (years)	10.0±2.6	11.7±3.0	T=-1.384(19)	0.182
Sex (male/female)	9/2	5/5	X^2^=1.169(1)	0.280
Threshold (dB HL)	L:47.0±13.0R: 52.2±18.1	L: 18.1±5.4R:19.4±4.9	Hearing loss: T=-0.847(10)Normal: T=-1.29(9)	0.4170.229

Note.—Data are mean ± standard deviation. L, left ear; R, right ear; HL, hearing level; df, degree of freedom. p<0.05 is considered as a significant difference.

No subjects were excluded because of excessive head movement (greater than 2.5 mm or 2.5 degree) during imaging.

### MRI data acquisition

MRI data of 21participants were acquired using a GE Discovery MR 750 3-T scanner. Each subject underwent an 8.06-minute scan during a conscious resting-state. Functional images were collected axially using an echo-planar imaging sequence (echo time (TE) = 2000 ms; repetition time (TR) = 30 ms; matrix size = 64 × 64 × 41, voxel size 3.4375 mm×3.4375 mm×3.2 mm). A three-dimensional T1-weighted structural image was also acquired for each subject using a magnetization prepared gradient echo sequence (TR =8.156 ms, TE =3.18 ms, 176 slices with 1 mm×1 mm×1 mm voxels).

### Data preprocessing

Data were preprocessed using spm8 (http://www.fil.ion.ucl.ac.uk/spm/software/spm8/) and in-house C++ codes. Slice timing and motion corrections were first executed, and then processed functional images and structural ones were normalized to the standard brain template from the Montreal Neurological Institute (MNI 152) by applying nonlinear registration. The normalized images were smoothed using a 3D isotropic Gaussian kernel of full-width at half-maximum 8mm. Running segmentation for structural images generated white matter, gray matter, and cerebrospinal fluid images. Finally, the following procedures were applied: the removal of linear and quadratic trends; regress out covariates including six realignment parameters, white matter, and cerebrospinal fluid; band-pass temporal filter. To contain more frequency components, the frequency range of band-pass temporal filter was from 0.0039Hz to 0.1039Hz.

### Methods

Our method consists of two steps: (1) We constructed the individual specific virtual brain using resting-state fMRI data of each participant. These data had been preprocessed in the section data preprocessing. Each virtual brain comprised of an effective network that was constructed utilizing the entropy connectivity method [14], and every node in the network was corresponding to one Brodmann's Area (BA). The neural activity of each BA was calculated utilizing a multivariate regress model that was derived from the multiple linear regress model [26] and causal analysis method [14]. When a stimulating signal was exerted to one BA of the virtual brain, neural activities of all of BAs could be obtained by the multivariate regress model and an iterative method. (2) We exerted a stimulating signal to the primary auditory cortices of the virtual brain. Every virtual brain was constructed on the basis of resting-state fMRI data of each participant. Therefore, we could study changes of brain region activations in patients with long-term bilateral hearing loss by observing neural activities of brain regions in these virtual brains.

### Statistical analyses

The chi-squared test and two-sample t-test were performed to explore group differences in sex, age, educational levels, hearing levels, the activated strength of brain region, and neuropsychological testing scores. Statistical analyses were conducted with software (SPSS, version 19.0), and a P value of less than .05 was considered to indicate a significant difference for any single analysis.
